# Association of Early Aspirin Use With In-Hospital Mortality in Patients With Moderate COVID-19

**DOI:** 10.1001/jamanetworkopen.2022.3890

**Published:** 2022-03-24

**Authors:** Jonathan H. Chow, Ali Rahnavard, Mardi Gomberg-Maitland, Ranojoy Chatterjee, Pranay Patodi, David P. Yamane, Andrea R. Levine, Danielle Davison, Katrina Hawkins, Amanda M. Jackson, Megan T. Quintana, Allison S. Lankford, Ryan J. Keneally, Mustafa Al-Mashat, Daniel Fisher, Jeffrey Williams, Jeffrey S. Berger, Michael A. Mazzeffi, Keith A. Crandall

**Affiliations:** 1 Department of Anesthesiology and Critical Care Medicine, George Washington University School of Medicine and Health Sciences, Washington, DC; 2 George Washington University Computational Biology Institute, Department of Biostatistics and Bioinformatics, Milken Institute School of Public Health, Washington, DC; 3 Division of Cardiology, Department of Medicine, George Washington University School of Medicine and Health Sciences, Washington, DC; 4 Department of Emergency Medicine, George Washington University School of Medicine and Health Sciences, Washington, DC; 5 Division of Pulmonary and Critical Care, Department of Medicine, University of Maryland School of Medicine, Baltimore; 6 Division of Gynecologic Oncology, Department of Obstetrics and Gynecology, Walter Reed National Military Medical Center, Bethesda, Maryland; 7 Department of Surgery, George Washington University School of Medicine and Health Sciences, Washington, DC; 8 Division of Maternal Fetal Medicine, Department of Obstetrics and Gynecology, University of Maryland School of Medicine, Baltimore; 9 Division of Critical Care Medicine, Department of Surgery, R. Adams Cowley Shock Trauma Center, Baltimore, Maryland

## Abstract

**Importance:**

Prior observational studies suggest that aspirin use may be associated with reduced mortality in high-risk hospitalized patients with COVID-19, but aspirin’s efficacy in patients with moderate COVID-19 is not well studied.

**Objective:**

To assess whether early aspirin use is associated with lower odds of in-hospital mortality in patients with moderate COVID-19.

**Design, Setting, and Participants:**

Observational cohort study of 112 269 hospitalized patients with moderate COVID-19, enrolled from January 1, 2020, through September 10, 2021, at 64 health systems in the United States participating in the National Institute of Health’s National COVID Cohort Collaborative (N3C).

**Exposure:**

Aspirin use within the first day of hospitalization.

**Main Outcome and Measures:**

The primary outcome was 28-day in-hospital mortality, and secondary outcomes were pulmonary embolism and deep vein thrombosis. Odds of in-hospital mortality were calculated using marginal structural Cox and logistic regression models. Inverse probability of treatment weighting was used to reduce bias from confounding and balance characteristics between groups.

**Results:**

Among the 2 446 650 COVID-19–positive patients who were screened, 189 287 were hospitalized and 112 269 met study inclusion. For the full cohort, Median age was 63 years (IQR, 47-74 years); 16.1% of patients were African American, 3.8% were Asian, 52.7% were White, 5.0% were of other races and ethnicities, 22.4% were of unknown race and ethnicity. In-hospital mortality occurred in 10.9% of patients. After inverse probability treatment weighting, 28-day in-hospital mortality was significantly lower in those who received aspirin (10.2% vs 11.8%; odds ratio [OR], 0.85; 95% CI, 0.79-0.92; *P* < .001). The rate of pulmonary embolism, but not deep vein thrombosis, was also significantly lower in patients who received aspirin (1.0% vs 1.4%; OR, 0.71; 95% CI, 0.56-0.90; *P* = .004). Patients who received early aspirin did not have higher rates of gastrointestinal hemorrhage (0.8% aspirin vs 0.7% no aspirin; OR, 1.04; 95% CI, 0.82-1.33; *P* = .72), cerebral hemorrhage (0.6% aspirin vs 0.4% no aspirin; OR, 1.32; 95% CI, 0.92-1.88; *P* = .13), or blood transfusion (2.7% aspirin vs 2.3% no aspirin; OR, 1.14; 95% CI, 0.99-1.32; *P* = .06). The composite of hemorrhagic complications did not occur more often in those receiving aspirin (3.7% aspirin vs 3.2% no aspirin; OR, 1.13; 95% CI, 1.00-1.28; *P* = .054). Subgroups who appeared to benefit the most included patients older than 60 years (61-80 years: OR, 0.79; 95% CI, 0.72-0.87; *P* < .001; >80 years: OR, 0.79; 95% CI, 0.69-0.91; *P* < .001) and patients with comorbidities (1 comorbidity: 6.4% vs 9.2%; OR, 0.68; 95% CI, 0.55-0.83; *P* < .001; 2 comorbidities: 10.5% vs 12.8%; OR, 0.80; 95% CI, 0.69-0.93; *P* = .003; 3 comorbidities: 13.8% vs 17.0%, OR, 0.78; 95% CI, 0.68-0.89; *P* < .001; >3 comorbidities: 17.0% vs 21.6%; OR, 0.74; 95% CI, 0.66-0.84; *P* < .001).

**Conclusions and Relevance:**

In this cohort study of US adults hospitalized with moderate COVID-19, early aspirin use was associated with lower odds of 28-day in-hospital mortality. A randomized clinical trial that includes diverse patients with moderate COVID-19 is warranted to adequately evaluate aspirin’s efficacy in patients with high-risk conditions.

## Key Points

**Question:**

Is early aspirin use in hospitalized patients with moderate COVID-19 associated with lower odds of in-hospital mortality?

**Findings:**

In a cohort study of 112 269 patients with moderate COVID-19, early aspirin use during the first day of hospitalization was associated with lower 28-day in-hospital mortality and pulmonary embolism incidence when compared with patients who did not receive early aspirin.

**Meaning:**

This study suggests that early aspirin use may be associated with lower odds of in-hospital mortality among hospitalized patients with moderate COVID-19; these findings warrant further study in a randomized clinical trial that includes diverse patients with cardiovascular comorbidities.

## Teaser

This cohort study investigates whether early aspirin use is associated with lower 28-day in-hospital mortality among patients with moderate COVID-19.

## Introduction

SARS-CoV-2 continues to infect more than 22 million patients per week and globally causes more than 65 000 deaths per week as of January 31, 2022.^1^ Despite widespread vaccination efforts in wealthy countries, only 10% of the population in low-income countries is vaccinated.^2^ With low global vaccination rates and overwhelmed health systems, there continues to be a need for COVID-19 treatments, particularly in patients with moderate to severe disease.

Although aspirin has not been shown to reduce major adverse cardiovascular or pulmonary events in symptomatic outpatients with COVID-19, several observational studies have found lower risk-adjusted mortality in hospitalized patients who received aspirin.^3,4,5,6^ A large, propensity-matched cohort study found that prehospital antiplatelet therapy was associated with lower odds of mortality.^7^ In a randomized clinical trial (RCT) of aspirin-naive patients with few comorbidities, aspirin significantly increased the rate of being discharged alive within 28 days by 1.2% but did not reduce 28-day all-cause mortality.^8^ Limitations of that study included the relatively low prevalence of comorbidities, lower prevalence of obesity in the United Kingdom than in the United States, and lack of racial and ethnic diversity. For these reasons, additional studies examining the effects of aspirin in high-risk, diverse patient populations are necessary. We conducted the Analysis of National COVID-19 Hospitalization Outcomes in Recipients of Aspirin (ANCHOR) study to assess whether early aspirin use was associated with lower odds of mortality in patients with moderate COVID-19. Our hypothesis was that early aspirin use would be associated with lower odds of 28-day in-hospital mortality.

## Methods

### Study Design

For this cohort study, patients were identified using the National Institutes of Health’s National COVID Cohort Collaborative (N3C) Data Enclave.^9^ Patients were included if they were hospitalized with a diagnosis of SARS-CoV-2 confirmed by polymerase chain reaction or antigen test results and if they met criteria for moderate COVID-19 severity on the first day of hospitalization. Patients who were not hospitalized or who met criteria for severe COVID-19 on the first day of hospitalization were excluded from the study.

The COVID-19 severity level was based on the World Health Organization Clinical Progression Scale. We defined mild disease as asymptomatic or symptomatic infection not requiring hospitalization, moderate disease as infection requiring hospitalization, and severe disease as infection causing death or the need for invasive mechanical ventilation, vasopressors, inotropes, or extracorporeal membrane oxygenation. The study was exempted by the institutional review board at the George Washington University under 45 CFR 46.101(b), and for this reason informed written consent was waived. The N3C data transfer to the National Center for Advancing Translational Sciences was performed under Johns Hopkins University Reliance Protocol IRB00249128 and individual site agreements with the National Institutes of Health. The authors used the Strengthening the Reporting of Observational Research Studies in Epidemiology (STROBE) guideline for cohort studies to confirm appropriate methodology and reporting for this study.

### Data Collection

The N3C Data Enclave contained data from 7 930 729 patients from 64 health systems in the United States. The data underwent acquisition from participating hospitals, harmonization, validation, centralized mapping to the Observational Medical Outcomes Partnership 5.3.1 vocabulary, and deidentification and then were made available to each institution.^9,10^ Patients were enrolled starting January 1, 2020, and the data set was frozen on September 10, 2020. The data set consisted of 2 446 850 COVID-19–positive patients, 4.1 billion laboratory results, 1.3 billion drug exposures, and 406 million health care encounters. Clinical data, including comorbidities, medications, laboratory values, and outcome data, were identified using existing concept identification numbers in the Observational Medical Outcomes Partnership's common data model.

### Primary, Secondary, and Safety Outcomes

Early aspirin use was defined as the administration of aspirin as part of routine care within the first day of hospitalization. The primary outcome was in-hospital 28-day mortality, and secondary outcomes included in-hospital acute pulmonary embolism (PE) and acute deep vein thrombosis (DVT). We hypothesized that patients with moderate COVID-19 receiving early aspirin would have a significantly lower rate of in-hospital mortality, PE, and DVT. Hemorrhagic complications, such as gastrointestinal hemorrhage, cerebral hemorrhage, blood transfusion (defined as red blood cell or whole blood transfusion), and the composite of hemorrhagic complications, were also compared between groups as safety outcomes.

### Statistical Analysis

Data acquisition and analysis was performed using Palantir Foundry (Palantir Technologies Inc) and R, version 4.0.2 (R Foundation for Statistical Computing) using the ipw and survey packages.^11,12^ Continuous variables with a nonparametric distribution were summarized as median and IQR. Categorical variables were summarized as the number and percentage of patients.

To reduce confounding, we performed inverse probability of treatment weighting (IPTW) with stabilized weights.^13,14^ Inverse probability of treatment weighting allows for estimation of the mean treatment effect if there is no unmeasured confounding and the propensity score is correctly specified.^15^ The IPTWs were calculated using propensity scores from a logistic regression model.^16^

For the logistic regression–propensity score model, aspirin was modeled as the dependent variable and independent variables were age, sex, race and ethnicity, chronic kidney disease, chronic obstructive pulmonary disease, asthma, heart disease, hypertension, diabetes, and history of aspirin use in the preceding 90 days. In addition, receipt of other therapeutics initiated on or prior to the first day of hospitalization, such as dexamethasone, remdesivir, tocilizumab, therapeutic heparin, and enoxaparin, were included as independent variables. A robust sandwich variance estimator was used to account for the estimated treatment weights and within-patient associations that are created with IPTW.^15,17^ This method leads to larger standard errors, resulting in more conservative estimates of the mean treatment effect and making it more difficult to reject the null hypothesis.

Balance in baseline covariates was assessed in the unweighted and weighted populations by calculating standardized mean differences (SMDs) for each covariate. An SMD of 0.2 or less was used as the threshold for a variable to be considered adequately balanced.^18^ The primary outcome was analyzed using a marginal structural Cox and logistic regression model with IPTWs. To compare differences in secondary outcomes, marginal structural logistic regressions were applied using the same weights. For the primary outcome, a number needed to treat was calculated from the absolute reduction in mortality. A 2-sided *P* value less than .05 was considered statistically significant for the primary outcome. For the 2 secondary outcomes, a Bonferroni correction was applied to account for multiple comparisons, and a *P* value less than .025 was considered statistically significant. An additional sensitivity analysis with an E-value was used to measure the amount of confounding that could mitigate the results.

#### Subgroup Analyses

Additional prespecified subgroup analyses were performed to examine the effect of early aspirin on the following groups: age (≤60 years vs >60 years) and number of comorbidities (0 vs ≥1). For each subgroup, the significance of the interaction between the treatment and the subgroup was tested. To account for multiplicity of the subgroups, a *P* value less than .025 was considered statistically significant. Because IPTW balances covariates across the overall population rather than across subgroups, subgroup analysis can lead to covariate imbalance when estimating the mean treatment effect within a subgroup.^19^ Therefore, as an additional sensitivity analysis, subgroup balancing was performed whereby IPTW was performed for every level within a subgroup to ensure adequate covariate balance.

#### Missing Data

Among the covariates used in the IPTW, the only independent variable with missing data was race and ethnicity. Because this may not have been missing at random, a separate “unknown” category of race and ethnicity was created. Other variables, such as body mass index (BMI, calculated as weight in kilometers divided by height in meters squared), vital signs, and laboratory values, were not included in the IPTW because of the high rate of missing data and were not imputed.

## Results

**Figure 1.**
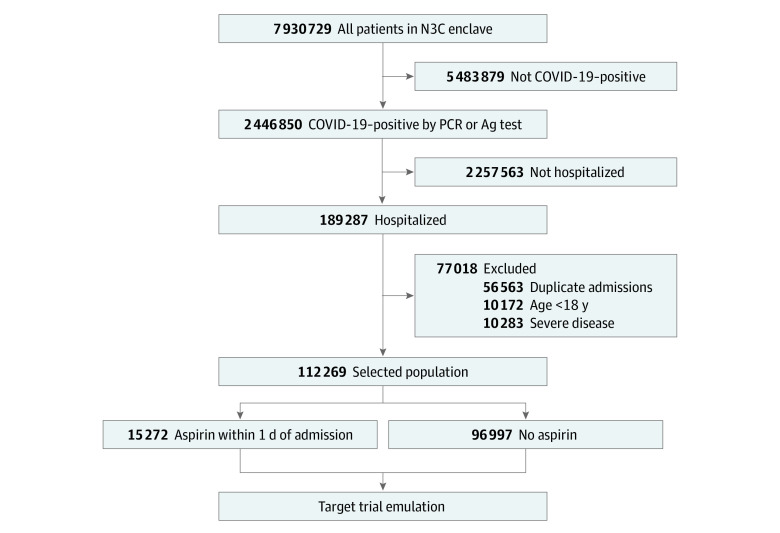
Flow Diagram Depicting the Phases of Enrollment, Exclusion, and Data Analysis Ag indicates antigen; N3C, National COVID Cohort Collaborative; and PCR, polymerase chain reaction.

A total of 7 930 729 patients were assessed (Figure 1). Of these, 2 446 850 were diagnosed as having COVID-19, and 189 287 were hospitalized. Duplicate admissions, patients younger than 18 years, and patients with severe disease on first day of hospitalization were removed, which yielded a final data set of 112 269 hospitalized patients with moderate COVID-19. For the full cohort, median age was 63 years (IQR, 47-74 years), and 16.1% of patients were African American, 3.8% were Asian, 22.4% were of unknown race and ethnicity, 52.7% were White, and 5.0% were of other races (the category “other” race includes those classified with multiple categorizations of race and those recorded with “other” race in the database). In-hospital mortality occurred in 10.9% of patients in the overall cohort. A total of 15 272 patients (13.6%) received aspirin in the first day of admission, while 96 997 (86.4%) did not (Table 1). Median aspirin dose was 81 mg (IQR, 81-81 mg), and median treatment duration was 5 days (IQR, 2-10 days). Among patients in the no aspirin group, receipt of aspirin in subsequent hospital days occurred in 3203 patients (3.1%). The median number of days from hospitalization to crossover was 4 days (IQR, 2-7 days). Prior to IPTW, patients in the aspirin group had higher rates of chronic kidney disease (39.4% vs 17.3%), chronic obstructive pulmonary disease (17.6% vs 10.3%), heart disease (55.3% vs 21.1%), hypertension (75.6% vs 43.9%), and diabetes (51.1% vs 27.2%). In addition, more patients in the aspirin group had a history of prior aspirin use than patients in the nonaspirin group (46.9% vs 4.2%).

**Table 1.** Baseline Demographic and Other Characteristics Before and After IPTW^a^

Variable	Before IPTW, No. (%)^b^			After IPTW, No. (%)^c^		
	No aspirin (n = 96 997)	Aspirin (n = 15 272)	SMD	No aspirin (n = 98 275)	Aspirin (n = 13 795)	SMD
Demographic characteristics						
Age, median (IQR), y	61 (44-73)	69 (60-77)	0.53	63 (47-75)	65 (53-74)	0.12
Sex						
Male	47 216 (48.7)	9000 (58.9)	0.21	49 280 (50.1)	7018 (50.9)	0.02
Female	49 781 (51.3)	6272 (41.1)		48 996 (49.9)	6777 (49.1)	
BMI	28.9 (23.6-35.4)	29.2 (25.0-35.4)	NA	28.8 (23.6-35.3)	29.5 (25.0-36.0)	NA
Race and ethnicity^d^						
African American	14 848 (15.3)	3249 (21.3)	0.30	15 045 (15.3)	3035 (22.0)	0.19
Asian/Pacific Islander	3462 (3.6)	792 (5.2)		3573 (3.6)	651 (4.7)	
Unknown	23 048 (23.8)	2110 (13.8)		21 791 (22.2)	2794 (20.3)	
White	50 579 (52.1)	8564 (56.1)		52 843 (53.8)	6764 (49.0)	
Other	5060 (5.2)	557 (3.6)		5023 (5.1)	551 (4.0)	
Comorbidities						
Chronic kidney disease	16 819 (17.3)	6020 (39.4)	0.51	20 421 (20.8)	3267 (23.7)	0.07
COPD	10 026 (10.3)	2689 (17.6)	0.21	11 298 (11.5)	1762 (12.8)	0.04
Asthma	9127 (9.4)	1843 (12.1)	0.09	9659 (9.8)	1423 (10.3)	0.02
Heart disease	20 421 (21.1)	8439 (55.3)	0.75	26128 (26.6)	4093 (29.7)	0.07
Hypertension	42 574 (43.9)	11 539 (75.6)	0.68	48 021 (48.9)	7386 (53.5)	0.09
Diabetes	26 374 (27.2)	7807 (51.1)	0.51	30 385 (30.9)	47 13 (34.2)	0.07
Prior aspirin use	4063 (4.2)	7164 (46.9)	1.12	10 947 (11.1)	1583 (11.5)	0.01
Admission vital signs^a^						
Blood pressure, mm Hg						
Systolic	103 (94-114)	103 (93-115)	NA	103 (94-114)	105 (95-115)	NA
Diastolic	58 (51-65)	56 (49-63)	NA	57 (50-65)	58 (51-65)	NA
HR, bpm	104 (93-117)	100 (89-114)	NA	104 (93-117)	100 (89-113)	NA
RR, per min	23 (20-29)	25 (21-31)	NA	23 (20-30)	24 (20-30)	NA
Spo_2_, %	92 (87-95)	91 (86-93)	NA	92 (87-95)	92 (88-94)	NA
Temperature, °C	37.4 (37.0-38.1)	37.4 (37.0-38.2)	NA	37.4 (37.0-38.1)	37.4 (37.0-38.1)	NA
Initial laboratory values^a^						
WBC, K/μL	8.1 (5.9-11.8)	8.2 (5.9-11.4)	NA	8.2 (6.0-12.0)	7.8 (5.6-11.0)	NA
Lymphocytes, K/μL	1.0 (0.7-1.2)	0.8 (0.5-1.2)	NA	1.0 (0.6-1.2)	0.9 (0.6-1.4)	NA
Platelets, K/μL	193 (147-250)	186 (142-243)	NA	191 (145-249)	196 (150-252)	NA
INR	1.1 (1.0-1.3)	1.1 (1.0-1.3)	NA	1.1 (1.0-1.3)	1.1 (1.0-1.3)	NA
Fibrinogen, mg/dL	525 (410-646)	536 (418-655)	NA	525 (410-646)	528 (407-650)	NA
Lactate, mg/dL	12.6 (9.0-18.0)	14.4 (9.9-19.8)	NA	13.5 (9.0-18.9)	13.5 (9.9-19.8)	NA
Pao_2_, mm Hg	90 (78-95)	90 (84-93)	NA	90 (78-94)	91 (86-94)	NA
Therapeutic agents initiated by first day of hospitalization						
Dexamethasone	16 146 (16.6)	5795 (37.9)	0.49	20 298 (20.7)	3141 (22.8)	0.05
Remdesivir	9253 (9.5)	2513 (16.5)	0.21	10 619 (10.8)	1584 (11.5)	0.02
Tocilizumab	315 (0.3)	97 (0.6)	0.05	371(0.4)	64 (0.5)	0.01
Therapeutic heparin	1141 (1.2)	1217 (8.0)	0.33	2560 (2.6)	375 (2.7)	0.01
Enoxaparin	5390 (5.6)	1855 (12.1)	0.23	6539 (6.7)	1095 (7.9)	0.05

Abbreviations: BMI, body mass index (calculated as weight in kilograms divided by height in meters squared); BP, blood pressure; COPD, chronic obstructive pulmonary disease; HR, heart rate; INR, international normalized ratio; IPTW, inverse probability of treatment weighting; NA, not applicable; Pao_2_, partial pressure of arterial oxygen; RR, respiratory rate; SMD, standardized mean difference; Spo_2_, peripheral capillary oxygen saturation; WBC, white blood cell count.

SI conversion factors: To convert the values for fibrinogen to g/L, multiply by 0.01; lactate to mmol/L, multiply by 0.111; lymphocytes and WBC count to ×10^9^ per liter, multiply by 0.001; and platelets to ×10^9^ per liter, multiply by 1.

^a^ Data were missing for a substantial proportion of patients and therefore not included in the IPTW analysis for BMI (60.6%), systolic BP (73.1%), diastolic BP (73.3%), HR (75.3%), RR (79.1%), Spo_2_ (72.3%), temperature (77.7%), WBC (17.4%), lymphocytes (39.2%), platelets (11.2%), INR (61.7%), fibrinogen (84.6%), lactate (68.6%), and Pao_2_ (70.8%). When multiple measurements were recorded during the first day of hospitalization, the worst value was recorded.

^b^ Data are given as number (percentage) unless otherwise stated.

^c^ Data are given as number (percentage) unless otherwise stated. All values were generated after inverse probability of treatment weighting was performed and are therefore weighted.

^d^ Identification provided by database and electronic health record. “Other” includes those with multiple categorizations and those recorded with “other” in the database.

### Covariate Balance Before and After IPTW

The SMDs before IPTW were greater than 0.2 in 12 of 15 covariates (80%) (eFigure in Supplement 1). After IPTW was performed (stabilized mean [SD] weight, 0.998 [0.657]), baseline demographic characteristics, comorbidities, and receipt of therapeutic treatments were similar between the groups, with all covariates having an SMD of 0.2 or less.

### Primary and Secondary Outcomes

**Figure 2.**
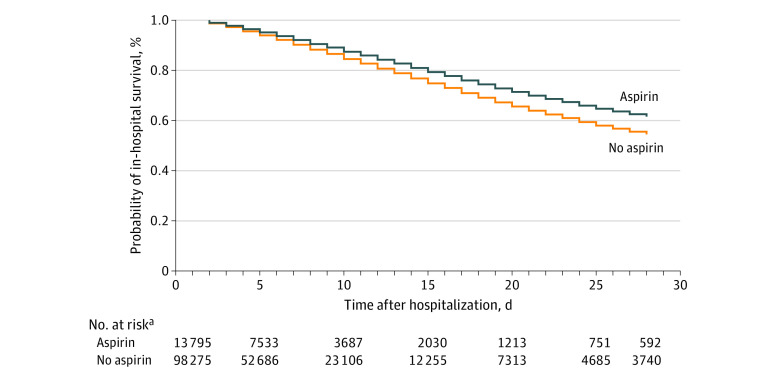
In-Hospital Survival at 28 Days Survival function in patients receiving aspirin and not receiving aspirin. Patients discharged within the study period are right-censored. In patients with moderate disease on hospital admission, aspirin use was associated with increased survival (adjusted hazard ratio, 0.80; 95% CI, 0.74-0.86; *P* < .001). ^a^All values were generated after inverse probability of treatment weighting was performed and are therefore weighted.

In-hospital 28-day mortality was significantly lower in the aspirin group (10.2% aspirin vs 11.8% no aspirin), resulting in significantly lower risk-adjusted mortality (odds ratio [OR], 0.85; 95% CI, 0.79-0.92; *P* < .001) (Figure 2). The relative mortality reduction of 13.6% and the absolute mortality reduction of 1.6% suggested that 63 patients would need to be treated with early aspirin to prevent 1 in-hospital death. In the sensitivity analysis with an E-value, we found that an unexplained confounder would need to be associated with both aspirin and in-hospital mortality at a risk ratio of 1.4 to make the hazard ratio equal to 1 and overturn the association between early aspirin and 28-day in-hospital mortality (E-value, 1.4; upper confidence limit, 1.3). The rate of in-hospital PE was significantly lower in the aspirin group (1.0% vs 1.4%; OR, 0.71; 95% CI, 0.56-0.90; *P* = .004), and DVT occurred in 1.0% of patients in both groups (OR, 1.00; 95% CI, 0.78-1.28; *P* = .98) (Table 2).

**Table 2.** Outcomes and Complications

Variable	Aspirin, No. (%) of patients^a^		Adjusted OR (95% CI)	*P* value
	No	Yes		
Primary outcome				
In-hospital mortality	11 577 (11.8)	1410 (10.2)	0.85 (0.79-0.92)	<.001
Secondary outcomes				
Pulmonary embolism	1355 (1.4)	136 (1.0)	0.71 (0.56-0.90)	.004
Deep vein thrombosis	1008 (1.0)	142 (1.0)	1.00 (0.78-1.28)	.98
Hemorrhagic complications^b^				
Gastrointestinal hemorrhage	730 (0.7)	107 (0.8)	1.04 (0.82-1.33)	.72
Cerebral hemorrhage	418 (0.4)	77 (0.6)	1.32 (0.92-1.88)	.13
Blood transfusion	2298 (2.3)	368 (2.7)	1.14 (0.99-1.32)	.06
Composite of hemorrhagic complications	3193 (3.2)	504 (3.7)	1.13 (1.00-1.28)	.054

Abbreviation: OR, odds ratio.

^a^ All values were generated after inverse probability of treatment weighting was performed and are therefore weighted.

^b^ Composite of hemorrhagic complications include gastrointestinal hemorrhage, cerebral hemorrhage, and blood transfusion.

### Hemorrhagic Complications

There were no significant differences in the rate of gastrointestinal hemorrhage (0.8% aspirin vs 0.7% no aspirin; OR, 1.04; 95% CI, 0.82-1.33; *P* = .72), cerebral hemorrhage (0.6% aspirin vs 0.4% no aspirin; OR, 1.32; 95% CI, 0.92-1.88; *P* = .13), or blood transfusion (2.7% aspirin vs 2.3% no aspirin; OR, 1.14; 95% CI, 0.99-1.32; *P* = .06). The composite of hemorrhagic complications did not occur more often in those receiving aspirin (3.7% aspirin vs 3.2% no aspirin; OR, 1.13; 95% CI, 1.00-1.28; *P* = .054).

### Subgroup Analysis

**Figure 3.**
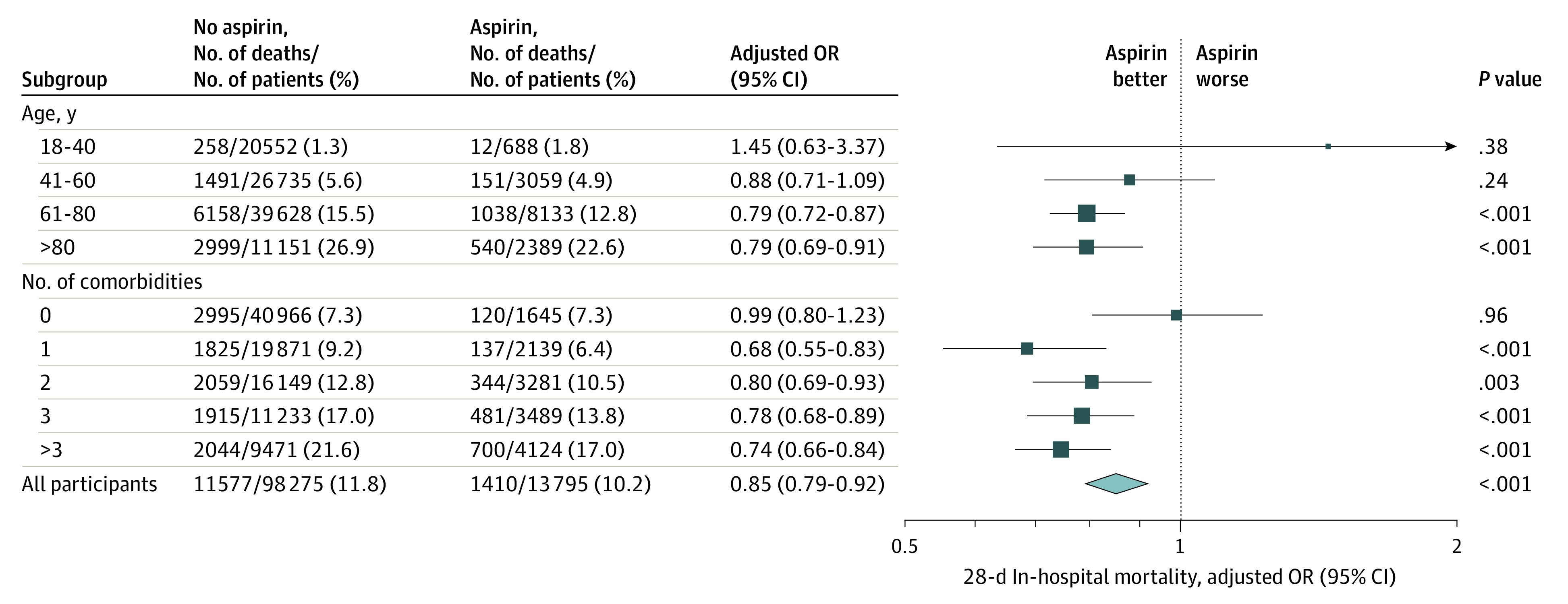
Subgroup Analyses Examining 28-Day In-Hospital Mortality After Early Aspirin Administration Shown are the prespecified subgroup analyses by age and number of comorbidities. The number of events, total number of patients, and event rate for each group are shown after inverse probability treatment weighting (IPTW). The odds ratio (OR) for all participants is plotted as a diamond, the ORs for each subgroup are plotted as squares, and the size of the squares is proportional to the standard error of the estimated effect size. The 95% CIs are plotted as horizontal lines. The right arrow indicates a CI that exceeds the limit of the x-axis. For the categories of age and number of comorbidities, an interaction term between the treatment and the category of interest was created. For age, this corresponds to a test of 60 years or younger vs older than 60 years (*P* = .001). For number of comorbidities, this corresponds to a test of 0 comorbidities vs at least 1 comorbidity (*P* < .001). As an additional sensitivity analysis, subgroup balancing was performed whereby IPTW was reperformed for every level within a subgroup (ie, aged 18-40, 41-60, 61-80 years, and >80 years) to ensure adequate covariate balance within subgroups. *P* values correspond to the significance of the OR difference from 1.

The association between early aspirin and decreased mortality was greater in patients older than 60 years (*F* statistic = 10.8; *P* for interaction = .001) and in patients with at least 1 comorbidity (*F* statistic = 20.2; *P* for interaction <.001). This was consistent after subgroup balancing, and patients receiving early aspirin between ages 18 and 40 years and 41 and 60 years did not have lower odds of mortality. However, patients between ages 61 and 80 years (OR, 0.79; 95% CI, 0.72-0.87; *P* < .001) and older than 80 years (OR, 0.79; 95% CI, 0.69-0.91; *P* < .001) receiving early aspirin had lower odds of mortality. In patients without comorbidities, there was no association between early aspirin and mortality (OR, 0.99; 95% CI, 0.80-1.23; *P* = .96), whereas in those with 1, 2, 3, and more than 3 comorbidities receiving early aspirin, there were lower odds of mortality (1 comorbidity: 6.4% vs 9.2%; OR, 0.68; 95% CI, 0.55-0.83; *P* < .001; 2 comorbidities: 10.5% vs 12.8%; OR, 0.80; 95% CI, 0.69-0.93; *P* = .003; 3 comorbidities: 13.8% vs 17.0%, OR, 0.78; 95% CI, 0.68-0.89; *P* < .001; >3 comorbidities: 17.0% vs 21.6%; OR, 0.74; 95% CI, 0.66-0.84; *P* < .001). (Figure 3).

## Discussion

In this cohort study of 112 269 patients with moderately severe COVID-19, treatment with early aspirin was associated with significantly lower odds of 28-day in-hospital mortality and PE. Despite the availability of effective vaccines in wealthy nations, COVID-19 continues to cause more than 65 000 deaths per week worldwide, highlighting the need for accessible, inexpensive therapies in those who are not vaccinated.^2^

### Prior Studies

Our findings are consistent with several prior observational studies of hospitalized patients.^3,4,7,20^ The first pilot study, the Collaborative Registry to Understand the Sequelae of Harm in COVID-19 (CRUSH-COVID),^3^ found that the composite exposure of prehospital and in-hospital aspirin use within the first 24 hours of admission was associated with decreased in-hospital mortality (adjusted hazard ratio, 0.53; 95% CI, 0.31-0.90; *P* = .02).^3^ In a study of 12 600 veterans, there were lower odds of 30-day all-cause mortality in those who received aspirin as an outpatient (OR, 0.38; 95% CI, 0.33-0.45).^4^ The COVID-19 Analysis to Assess the Mortality Impact of Antiplatelet Regimens at North American Centers (CATAMARAN) study^7^ found that, in 17 347 propensity-matched patients, prehospital antiplatelet therapy was associated with a 2.6% absolute reduction in mortality (adjusted hazard ratio, 0.81; 95% CI, 0.76-0.87; *P* < .005) and PE (2.2% antiplatelet therapy vs 3.0% no antiplatelet therapy; *P* = .002).^7^ These observational studies, although intriguing, could not establish causality and were prone to residual confounding.

The Randomised Evaluation of COVID-19 Therapy (RECOVERY) trial^8^ randomized 14 892 antiplatelet-naive patients to receive aspirin or placebo. In that study, all-cause 28-day mortality was not different between the 2 groups (rate ratio, 0.96; 95% CI, 0.89-1.04; *P* = .35). However, the proportion discharged alive within 28 days was higher in patients who received aspirin (74.8% aspirin vs 73.6% no aspirin; rate ratio, 1.06; 95% CI, 1.02-1.10; *P* = .006).^8^ Because the study excluded patients already receiving antiplatelet therapy prior to hospitalization, the rate of comorbidities was relatively low.^8^ Nevertheless, to our knowledge, this is the only large RCT to explore the efficacy of aspirin in patients hospitalized with COVID-19.

### Thrombotic Outcomes

Although median aspirin use in our study was 5 days, aspirin irreversibly inhibits cyclooxygenase for the 7- to 10-day life span of a platelet. Therefore, its antiplatelet effect was likely longer than the 5-day median duration of administration, which explains why such a short duration of aspirin use can have consequences for mortality. Similar to the CATAMARAN study,^7^ our study found that patients receiving early aspirin had lower odds of PE; however, we found no difference in odds of DVT. There are several possible explanations for these findings. First, this was a retrospective study where DVTs may have been underreported, so the nonsignificant difference may be a limitation of study ascertainment rather than a biologically meaningful result. Second, there may have been more frequent use of computed tomography angiography to assess for PE. Lower-yield Doppler ultrasonography studies may have been avoided to limit occupational exposure to COVID-19, especially in the absence of overt clinical signs of DVT. Third, while some of aspirin’s beneficial effect may occur through irreversible antiplatelet effects (cyclooxygenase 1 inhibition), aspirin also inhibits inducible cyclooxygenase 2, which decreases prostaglandin synthesis and propagation of anti-inflammatory signaling.^21,22^ It is possible that the mortality benefit of aspirin may be occurring through this pathway as well.

### Subgroup Analyses

Our subgroup analysis identified 2 groups that may benefit from early aspirin therapy. Patients older than 60 years had significantly lower odds of in-hospital mortality, whereas those who were younger did not. Given prior studies that have shown increased mortality with age, it is not surprising that aspirin’s efficacy may be greater in older patients.^23,24,25^ The number of comorbidities also appeared to modify aspirin’s association with mortality. Patients without comorbidities receiving early aspirin did not have lower odds of mortality, while those with at least 1 comorbidity had lower odds of mortality. Several studies have found an association between the number of comorbidities and mortality in COVID-19, and the larger effect size of early aspirin in these subgroups with comorbidities is consistent with this pattern.^25,26^

### Safety

This study did not find a significant difference in the rate of gastrointestinal hemorrhage, cerebral hemorrhage, or blood transfusion. Although numerically greater, the difference in the composite of hemorrhagic complications was not significantly higher in the early aspirin group. Antiplatelet medications carry significant risk, and the risk of hemorrhagic complications is well described in patients receiving aspirin for the primary prevention of cardiovascular disease. In RECOVERY, there was a 0.6% absolute increase in major bleeding events in those allocated to aspirin (1.6% aspirin vs 1.0% usual care; relative risk, 1.55; 95% CI, 1.16-2.07; *P* = .003). Multiple other studies have reported major hemorrhagic complications occurring at a rate 1.3 to 2.1 times higher in patients receiving aspirin for the primary prevention of cardiovascular disease.^27,28,29,30^ Because of this, the risk of bleeding complications should be carefully considered in every patient prior to the administration of antiplatelet medications.

### Limitations

Despite the large sample size and diversity of institutions contributing to our study, there are several important limitations. First, there may have been miscoding or underreporting of certain diagnoses (ie, bleeding complications) in the database, which is a limitation inherent to observational studies. Although these diagnoses are subject to underdetection, we believe that it is very unlikely that the underreporting of these diagnoses would be disproportionally different in the aspirin or no aspirin groups. Second, crossover from the control to the aspirin group occurred in 3.3% of patients, which may have biased our effect estimates toward the null hypothesis. Third, although data points were harmonized within the N3C Data Enclave, there were a substantial number of missing laboratory values and vital signs. In particular, there was a large proportion of missing BMI data; for this reason, obesity was not included as a covariate in the propensity score model. It is possible the exclusion of BMI from our model could bias our estimate effects, but BMI is also highly associated with many of the comorbidities that we balanced. Similarly, initial respiratory parameters such as oxygen flow and oxygen device were not consistently present, and it was difficult to ascertain the degree of respiratory insufficiency at baseline. Ideally, initial laboratory values and vital signs would have been used in the IPTW to more perfectly balance the aspirin and no aspirin groups, but this was not possible owing to the limitations of the data set. In addition, there was no information on do-not-resuscitate or do-not-intubate status, which may have affected the study’s outcomes. Fourth, this was not an RCT and causality cannot be established. It is possible that despite the inclusion of 15 covariates in our model, unmeasured confounders affected our results.

## Conclusions

In this observational cohort study of 112 269 hospitalized patients with moderate COVID-19, aspirin use in the first day of hospitalization was associated with lower odds of 28-day in-hospital mortality and PE. Important subgroups that may benefit from aspirin included patients older than 60 years and those with comorbidities. Although the composite of hemorrhagic complications was not significantly higher in the early aspirin group, aspirin’s risks must be carefully weighed before treatment. An RCT in a diverse patient population with high-risk conditions is needed to confirm our findings because our study cannot definitively establish causality.

## Notes

Supplement 1. **eFigure.** Standardized Mean Differences of Covariates Before and After IPTW

Supplement 2. **Nonauthor Collaborators.** N3C Consortium and ANCHOR Investigators

## References

[1] Johns Hopkins University. Coronavirus resource center. Published 2021. Accessed January 31, 2022. https://coronavirus.jhu.edu/

[2] Ritchie H, Mathieu E, Rodés-Guirao L, . Coronavirus pandemic (COVID-19). Published 2021. Accessed January 31, 2022. https://www.ourworldindata.org/coronavirus

[3] Chow JH, Khanna AK, Kethireddy S, . Aspirin use is associated with decreased mechanical ventilation, intensive care unit admission, and in-hospital mortality in hospitalized patients with coronavirus disease 2019. Anesth Analg. 2021;132(4):930-941. doi:10.1213/ANE.0000000000005292 33093359

[4] Osborne TF, Veigulis ZP, Arreola DM, Mahajan SM, Röösli E, Curtin CM. Association of mortality and aspirin prescription for COVID-19 patients at the Veterans Health Administration. PLoS One. 2021;16(2):e0246825. doi:10.1371/journal.pone.0246825 33571280 PMC7877611

[5] Meizlish ML, Goshua G, Liu Y, . Intermediate-dose anticoagulation, aspirin, and in-hospital mortality in COVID-19: a propensity score-matched analysis. Am J Hematol. 2021;96(4):471-479. doi:10.1002/ajh.26102 33476420 PMC8013588

[6] Connors JM, Brooks MM, Sciurba FC, ; ACTIV-4B Investigators. Effect of antithrombotic therapy on clinical outcomes in outpatients with clinically stable symptomatic COVID-19: the ACTIV-4B randomized clinical trial. JAMA. 2021;326(17):1703-1712. doi:10.1001/jama.2021.17272 34633405 PMC8506296

[7] Chow JH, Yin Y, Yamane DP, . Association of prehospital antiplatelet therapy with survival in patients hospitalized with COVID-19: a propensity score-matched analysis. J Thromb Haemost. 2021;19(11):2814-2824. doi:10.1111/jth.15517 34455688 PMC8646433

[8] RECOVERY Collaborative Group. Aspirin in patients admitted to hospital with COVID-19 (RECOVERY): a randomised, controlled, open-label, platform trial. Lancet. 2022;399(10320):143-151. doi:10.1016/S0140-6736(21)01825-0 34800427 PMC8598213

[9] Haendel MA, Chute CG, Bennett TD, ; N3C Consortium. The National COVID Cohort Collaborative (N3C): rationale, design, infrastructure, and deployment. J Am Med Inform Assoc. 2021;28(3):427-443. doi:10.1093/jamia/ocaa196 32805036 PMC7454687

[10] Pfaff ER, Girvin AT, Gabriel DL, ; N3C Consortium. Synergies between centralized and federated approaches to data quality: a report from the national COVID cohort collaborative. J Am Med Inform Assoc. 2021;ocab217. doi:10.1093/jamia/ocab217 34590684 PMC8500110

[11] Van der Wal WM, Geskus RB. Ipw: an R package for inverse probability weighting. J Stat Softw. 2011;43(13):1-23. doi:10.18637/jss.v043.i13

[12] Lumley T. Survey: analysis of complex survey samples. Published 2020. Accessed August 18, 2021. https://cran.r-project.org/web/packages/survey/

[13] Hernán MA, Robins JM. Using big data to emulate a target trial when a randomized trial is not available. Am J Epidemiol. 2016;183(8):758-764. doi:10.1093/aje/kwv254 26994063 PMC4832051

[14] Hernán MA, Sauer BC, Hernández-Díaz S, Platt R, Shrier I. Specifying a target trial prevents immortal time bias and other self-inflicted injuries in observational analyses. J Clin Epidemiol. 2016;79:70-75. doi:10.1016/j.jclinepi.2016.04.014 27237061 PMC5124536

[15] Austin PC, Stuart EA. Moving towards best practice when using inverse probability of treatment weighting (IPTW) using the propensity score to estimate causal treatment effects in observational studies. Stat Med. 2015;34(28):3661-3679. doi:10.1002/sim.6607 26238958 PMC4626409

[16] Hernan MA, Robins JM. Causal Inference: What If. Chapman & Hall/CRC; 2020.

[17] Schulte PJ, Mascha EJ. Propensity score methods: theory and practice for anesthesia research. Anesth Analg. 2018;127(4):1074-1084. doi:10.1213/ANE.0000000000002920 29750691

[18] Faraone SV. Interpreting estimates of treatment effects: implications for managed care. P T. 2008;33(12):700-703, 710-711. 19750051 PMC2730804

[19] Dong J, Zhang JL, Zeng S, Li F. Subgroup balancing propensity score. Stat Methods Med Res. 2020;29(3):659-676. doi:10.1177/0962280219870836 31456486

[20] Meizlish ML, Goshua G, Liu Y, . Intermediate-dose anticoagulation, aspirin, and in-hospital mortality in COVID-19: a propensity score-matched analysis. Am J Hematol. 2021;96(4):471-479. doi:10.1002/ajh.26102 33476420 PMC8013588

[21] Warner TD, Nylander S, Whatling C. Anti-platelet therapy: cyclo-oxygenase inhibition and the use of aspirin with particular regard to dual anti-platelet therapy. Br J Clin Pharmacol. 2011;72(4):619-633. doi:10.1111/j.1365-2125.2011.03943.x 21320154 PMC3195738

[22] Flower RJ. The development of COX2 inhibitors. Nat Rev Drug Discov. 2003;2(3):179-191. doi:10.1038/nrd1034 12612644

[23] Guan WJ, Ni ZY, Hu Y, ; China Medical Treatment Expert Group for Covid-19. Clinical characteristics of coronavirus disease 2019 in China. N Engl J Med. 2020;382(18):1708-1720. doi:10.1056/NEJMoa2002032 32109013 PMC7092819

[24] Ruan Q, Yang K, Wang W, Jiang L, Song J. Clinical predictors of mortality due to COVID-19 based on an analysis of data of 150 patients from Wuhan, China. Intensive Care Med. 2020;46(5):846-848. doi:10.1007/s00134-020-05991-x 32125452 PMC7080116

[25] Liang W, Liang H, Ou L, ; China Medical Treatment Expert Group for COVID-19. Development and validation of a clinical risk score to predict the occurrence of critical illness in hospitalized patients with COVID-19. JAMA Intern Med. 2020;180(8):1081-1089. doi:10.1001/jamainternmed.2020.2033 32396163 PMC7218676

[26] Zhou F, Yu T, Du R, . Clinical course and risk factors for mortality of adult inpatients with COVID-19 in Wuhan, China: a retrospective cohort study. Lancet. 2020;395(10229):1054-1062. doi:10.1016/S0140-6736(20)30566-3 32171076 PMC7270627

[27] McNeil JJ, Wolfe R, Woods RL, ; ASPREE Investigator Group. Effect of aspirin on cardiovascular events and bleeding in the healthy elderly. N Engl J Med. 2018;379(16):1509-1518. doi:10.1056/NEJMoa1805819 30221597 PMC6289056

[28] Ikeda Y, Shimada K, Teramoto T, . Low-dose aspirin for primary prevention of cardiovascular events in Japanese patients 60 years or older with atherosclerotic risk factors: a randomized clinical trial. JAMA. 2014;312(23):2510-2520. doi:10.1001/jama.2014.15690 25401325

[29] Gaziano JM, Brotons C, Coppolecchia R, ; ARRIVE Executive Committee. Use of aspirin to reduce risk of initial vascular events in patients at moderate risk of cardiovascular disease (ARRIVE): a randomised, double-blind, placebo-controlled trial. Lancet. 2018;392(10152):1036-1046. doi:10.1016/S0140-6736(18)31924-X 30158069 PMC7255888

[30] Bowman L, Mafham M, Wallendszus K, ; ASCEND Study Collaborative Group. Effects of aspirin for primary prevention in persons with diabetes mellitus. N Engl J Med. 2018;379(16):1529-1539. doi:10.1056/NEJMoa1804988 30146931

