# Supplementary material for: Association of Early Aspirin Use With In-Hospital Mortality in Patients With Moderate COVID-19
Source: JAMA Netw Open. 2022 Mar 24;5(3):e223890. doi: 10.1001/jamanetworkopen.2022.3890 (PMC8948531; doi:10.1001/jamanetworkopen.2022.3890)
Supplement: Supplement 1. — eFigure. Standardized Mean Differences of Covariates Before and After IPTW [file jamanetwopen-e223890-s001.pdf]

Supplementary Online Content

Chow JH, Rahnavard A, Gomberg-Maitland M, et al; N3C Consortium and ANCHOR Investigators. Association of early aspirin use with in-hospital mortality in patients with moderate COVID-19. *JAMA Netw Open*. 2022;5(3):e223890. doi:10.1001/jamanetworkopen.2022.3890

eFigure. Standardized Mean Differences of Covariates Before and After IPTW

This supplementary material has been provided by the authors to give readers additional information about their work.

© 2022 Chow JH et al. *JAMA Network Open*.

eFigure. Standardized Mean Differences of Covariates Before and After IPTW

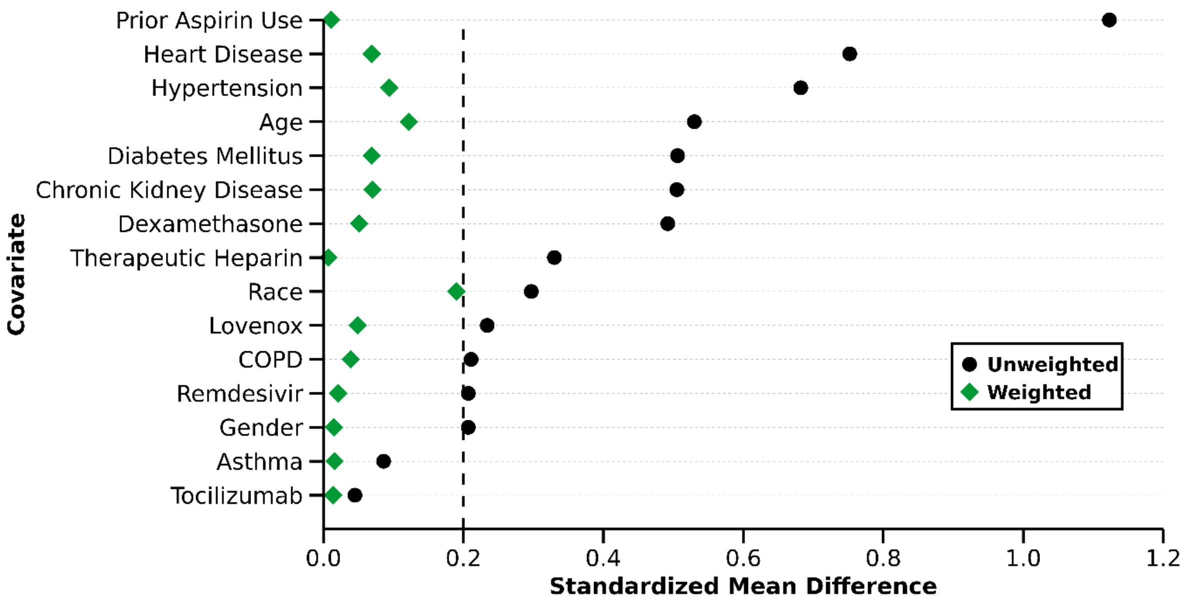

© 2022 Chow JH et al. *JAMA Network Open*.
