# Supplementary material for: Association of Early Aspirin Use With In-Hospital Mortality in Patients With Moderate COVID-19
Source: JAMA Netw Open. 2022 Mar 24;5(3):e223890. doi: 10.1001/jamanetworkopen.2022.3890 (PMC8948531; doi:10.1001/jamanetworkopen.2022.3890)
Supplement: Supplement 2. — Nonauthor Collaborators. N3C Consortium and ANCHOR Investigators [file jamanetwopen-e223890-s002.pdf]

Supplementary Online Material: Nonauthor Collaborators

*Indicates required information. Only first name, last name, and suffix will appear in PubMed.

*Group Name(s): N3C Consortium and ANCHOR Investigators

*First Name and Middle Initial(s)	*Last Name	*Suffix (eg, Jr, III)	Academic Degrees	Institution	Location (city, state/province, country)	Role or Contribution, eg, chair, principal investigator	Group (if more than 1 Group listed in the byline) and/or Subgroup (eg, Steering Committee)
Ivy	Benjenk		PhD	George Washington University School of Medicine and Health Sciences	Washington, DC, USA	Project Management, Data acquisition	ANCHOR
Benjamin	Amor		PhD	Palantir Technologies	Denver, CO, USA	Phenotype Team, Anal	N3C Consortium
Christopher	Austin		MD	National Institutes of Health	Bethesda, MD, USA	Partner from NIH and other federal agencies	N3C Consortium
Tellen	Bennett		MD	University of Colorado School of Medicine	Aurora, CO, USA	Workstream, subgrour	N3C Consortium
Mark	Bissell			Palantir Technologies	Denver, CO, USA	Phenotype Team, Anal	N3C Consortium
Samuel	Bozzette		MD, PhD	National Institutes of Health	Bethesda, MD, USA	Partner from NIH and other federal agencies	N3C Consortium
Katie	Bradwell		PhD	Palantir Technologies	Denver, CO, USA	Analytics Team	N3C Consortium
Carolyn	Bramante		MD, MPH	University of Minnesota	Minneapolis, MN, USA	Publication Committee	N3C Consortium
Yooree	Chae		MS	Sage Bionetworks	Seattle, WA, USA	Project Management and Operations Team	N3C Consortium
Christopher	Chute		MD, MPH	Johns Hopkins University	Baltimore, Maryland, USA	Workstream, subgrour	N3C Consortium
Marshall	Clark		BS	University of North Carolina at Chapel Hill	Chapel Hill, NC, USA	Phenotype Team	N3C Consortium
Conor	Cook		MA	Oregon Health & Science University	Portland, OR, USA	Project Management and Operations Team	N3C Consortium
Mariam	Deacy			National Institutes of Health	Bethesda, MD, USA	Partner from NIH and other federal agencies	N3C Consortium

*Indicates required information. Only first name, last name, and suffix will appear in PubMed.

***First Name and Middle Initial(s)**	***Last Name**	***Suffix (eg, Jr, III)**	Academic Degrees	Institution	Location (city, state/province, country)	Role or Contribution, eg, chair, principal investigator	Group (if more than 1 Group listed in the byline) and/or Subgroup (eg, Steering Committee)
Alexandra	Dest		MPH	Oregon Health & Science University	Portland, OR, USA	Project Management and Operations Team	N3C Consortium
Racquel	Dietz			Oregon Health & Science University	Portland, OR, USA	Project Management and Operations Team	N3C Consortium
Thomas	Dillon		BS	Washington University in St. Louis	St. Louis, MO, USA	Project Management and Operations Team	N3C Consortium
David	Eichmann		PhD	University of Iowa	Iowa City, IA, USA	Workstream, subgroup	N3C Consortium
Patricia	Francis			Johns Hopkins University School of Medicine	Baltimore, Maryland, USA	Project Management and Operations Team	N3C Consortium
Rafel	Fuentes			National Center for Advancing Translational Sciences	Bethesda, MD, USA	Project Management and Operations Team	N3C Consortium
Davera	Gabriel		RN	Johns Hopkins University School of Medicine	Baltimore, Maryland, USA	Clinical data model exp	N3C Consortium
Nicole	Garbarini		MD	Johns Hopkins University School of Medicine	Baltimore, Maryland, USA	Partner from NIH and other federal agencies	N3C Consortium
Kenneth	Gersing		MD	National Institutes of Health	Bethesda, MD, USA	Workstream, subgroup	N3C Consortium
Andrew	Girvin		PhD	Palantir Technologies	Denver, CO, USA	Phenotype Team, Anal	N3C Consortium
Alexis	Graves		MA	The University of Iowa	Iowa City, IA, USA	Project Management and Operations Team	N3C Consortium
Justin	Guinney		PhD	Sage Bionetworks	Seattle, WA, USA	Workstream, subgroup	N3C Consortium
Melissa	Haendel		PhD	Oregon Health & Science University	Portland, OR, USA	Funding acquisition, go	N3C Consortium
Jeremy	Harper		MBI	Owl Health Networks	Indianapolis, IN, USA	Publication Committee	N3C Consortium

Supplementary Online Material: Nonauthor Collaborators

*Indicates required information. Only first name, last name, and suffix will appear in PubMed.

*First Name and Middle Initial(s)	*Last Name	*Suffix (eg, Jr, III)	Academic Degrees	Institution	Location (city, state/province, country)	Role or Contribution, eg, chair, principal investigator	Group (if more than 1 Group listed in the byline) and/or Subgroup (eg, Steering Committee)
Wenndy	Hernandez			The University of Chicago	Chicago, IL, USA	Publication Committee	N3C Consortium
Stephanie	Hong		BS	Johns Hopkins University School of Medicine	Baltimore, Maryland, USA	Data curation, data int	N3C Consortium
Warren	Kibbe		PhD	Duke University	Durham, NC, USA	Workstream, subgroup	N3C Consortium
Farrukh	Koraishy		MD, PhD	Stony Brook University	Stony Brook, NY, USA	Publication Committee	N3C Consortium
Kristin	Kostka		MPH	Real World Solutions, IQVIA	Cambridge, MA, USA	Data Ingest and Harmonization Team, Phenotype Team	N3C Consortium
Michael	Kurilla		MD, PhD	National Center for Advancing Translational Sciences	Bethesda, MD, USA	Partner from NIH and other federal agencies	N3C Consortium
Adam	Lee		MBA	University of North Carolina at Chapel Hill	Chapel Hill, NC, USA	Phenotype Team	N3C Consortium
Harold	Lehmann		MD	Johns Hopkins University School of Medicine	Baltimore, Maryland, USA	Data Ingest and Harmonization Team	N3C Consortium
Hongfang	Liu		PhD	Mayo Clinic	Rochester, MN, USA	Workstream, subgroup	N3C Consortium
Amin	Manna		MS	Palantir Technologies	Denver, CO, USA	Analytics Team	N3C Consortium
Federico	Mariona			William Beaumont Hospital	Southfield, MI, USA	Publication Committee	N3C Consortium
Julie	McMurry		MPH	Oregon State University	Corvallis, OR, USA	Project Management and Operations Team, Publication Committee	N3C Consortium
Sam	Michael			National Center for Advancing Translational Sciences	Bethesda, MD, USA	Partner from NIH and other federal agencies	N3C Consortium
Robert	Miller					Phenotype Team	N3C Consortium

Supplementary Online Material: Nonauthor Collaborators

*Indicates required information. Only first name, last name, and suffix will appear in PubMed.

***First Name and Middle Initial(s)**	***Last Name**	***Suffix (eg, Jr, III)**	Academic Degrees	Institution	Location (city, state/province, country)	Role or Contribution, eg, chair, principal investigator	Group (if more than 1 Group listed in the byline) and/or Subgroup (eg, Steering Committee)
Richard	Moffitt		PhD	Stony Brook University	Stony Brook, NY, USA	Data Ingest and Harmonization Team	N3C Consortium
Michele	Morris		BA	University of Pittsburgh	Pittsburgh, PA, USA	Data Ingest and Harmonization Team, Phenotype Team	N3C Consortium
Andrew	Neumann		MBA	Oregon State University	Corvallis, OR, USA	Project Management and Operations Team	N3C Consortium
Shawn	O'Neil			Oregon State University	Corvallis, OR, USA	Project Management and Operations Team	N3C Consortium
Matvey	Palchuk		MD	TriNetX	Cambridge, MA, USA	Data Ingest and Harmonization Team, Phenotype Team	N3C Consortium
Philip	Payne		PhD	Washington University in St. Louis	St. Louis, MO, USA	Workstream, subgroup	N3C Consortium
Emily	Pfaff		PhD	University of North Carolina at Chapel Hill	Chapel Hill, NC, USA	Workstream, subgroup	N3C Consortium
Nabeel	Qureshi		BA	Palantir Technologies	Denver, CO, USA	Analytics Team	N3C Consortium
Peter	Robinson		MD	Jackson Laboratory	Bar Harbor, ME, USA	Workstream, subgroup	N3C Consortium
Joni	Rutter		PhD	National Center for Advancing Translational Sciences	Bethesda, MD, USA	Partner from NIH and other federal agencies	N3C Consortium
Joel	Saltz		MD	Stony Brook University	Stony Brook, NY, USA	Workstream, subgroup	N3C Consortium
Mary	Saltz		MD	Stony Brook University	Stony Brook, NY, USA	Publication Committee	N3C Consortium
Amit	Saha			Wake Forest Baptist Medical	Winston Salem, NC, USA	Publication Committee	N3C Consortium
Usman	Sheikh			National Center for Advancing Translational Sciences	Bethesda, MD, USA	Project Management and Operations Team	N3C Consortium

Supplementary Online Material: Nonauthor Collaborators

*Indicates required information. Only first name, last name, and suffix will appear in PubMed.

*First Name and Middle Initial(s)	*Last Name	*Suffix (eg, Jr, III)	Academic Degrees	Institution	Location (city, state/province, country)	Role or Contribution, eg, chair, principal investigator	Group (if more than 1 Group listed in the byline) and/or Subgroup (eg, Steering Committee)
Heidi	Spratt		PhD	University of Texas Medical Branch	Galveston, TX, USA	Workstream, subgroup	N3C Consortium
Justin	Starren		MD, PhD	Northwestern University	Evanston, IL, USA	Workstream, subgroup	N3C Consortium
Christine	Suver		PhD	Sage Bionetworks	Seattle, WA, USA	Workstream, subgroup	N3C Consortium
Meredity	Temple-O'Connor		PhD	National Center for Advancing Translational Sciences	Bethesda, MD, USA	Partner from NIH and other federal agencies	N3C Consortium
Satyanarayana	Vedula			Johns Hopkins University	Baltimore, Maryland, USA	Publication Committee	N3C Consortium
Andrea	Volz		BS	Oregon Health & Science University	Portland, OR, USA	Project Management and Operations Team, Publication Committee	N3C Consortium
Anita	Walden		MS	Oregon Health & Science University	Portland, OR, USA	Workstream, subgroup	N3C Consortium
Kellie	Walters		MPH	University of North Carolina at Chapel Hill	Chapel Hill, NC, USA	Phenotype Team	N3C Consortium
Adam	Wilcox		MPH	Washington University in St. Louis	St. Louis, MO, USA	Workstream, subgroup	N3C Consortium
Andew	Williams		PhD	Tufts Medical Center	Boston, MA, USA	Workstream, subgroup	N3C Consortium
Chunlei	Wu		PhD	Scripps Research	La Jolla, CA, USA	Workstream, subgroup	N3C Consortium
Elizabeth	Zampino		PhD	University of Washington	Seattle, WA, USA	Project Management and Operations Team	N3C Consortium
Ziaohan	Zhang		MD, MPH	Johns Hopkins University School of Medicine	Baltimore, Maryland, USA	Data Ingest and Harmonization Team	N3C Consortium
Richard	Zhu		MD	Johns Hopkins University School of Medicine	Baltimore, Maryland, USA	Data Ingest and Harmonization Team	N3C Consortium
